# Analysis of early failure rate and its risk factor with 2157 total ankle replacements

**DOI:** 10.1038/s41598-021-81576-y

**Published:** 2021-01-21

**Authors:** Jung Woo Lee, Woo-Young Im, Si Young Song, Jae-Young Choi, Sung Jae Kim

**Affiliations:** 1 Department of Orthopaedic Surgery, Yonsei University Wonju College of Medicine, Wonju Severance Christian Hospital, 20, Ilsan-ro, Wonju-si, Gangwon-do, 26426 Republic of Korea; 2 Department of Orthopaedic Surgery, Hallym University Dongtan Sacred Heart Hospital, 7, Keunjaebong-gil, Hwaseong-si, Gyeonggi-do, 18450 Republic of Korea; 3 School of Advanced Materials Science and Engineering, Sungkyunkwan University, 2066, Seobu-ro, Jangan-gu, Suwon-si, Gyeonggi-do 16419 Republic of Korea; 4 SKKU Advanced Institute of Nanotechnology (SAINT), Sungkyunkwan University, Suwon-si, Republic of Korea

**Keywords:** Risk factors, Outcomes research

## Abstract

The failure rate of TAA is still higher than that of other joint replacement procedures. This study aimed to calculate the early failure rate and identify associated patient factors. Data from the Korean Health Insurance Review and Assessment Service database from 2009 to 2017 were collected. We evaluated patients who had TAA as a primary surgical procedure. Early failure was defined as conversion to revision TAA or arthrodesis after primary TAA within five years. Patients with early failure after primary TAA were designated as the “Failure group”. Patients without early failure and who were followed up unremarkably for at least five years after primary TAA were designated as the “No failure group”. Overall, 2157 TAA participants were included. During the study period, 197 patients developed failure within five years postoperatively, for an overall failure rate of 9.1%. Significant risk factors for early failure were history of chronic pulmonary disease, diabetes, peripheral vascular disease, hyperlipidemia, dementia, and alcohol abuse. A significant increase of odds ratio was found in patients with a history of dementia, chronic pulmonary disease, and diabetes. Surgical indications and preoperative patient counseling should consider these factors.

## Introduction

Total ankle arthroplasty (TAA) is growing in popularity along with ankle arthrodesis for the treatment of end-stage ankle arthritis^1^. An advantage of TAA includes maintaining mobility at the ankle joint and decreasing the radiographic incidence of adjacent joint degeneration, which is otherwise seen in ankle arthrodesis^2^. Improvements in implant design and technique have resulted in good short- and mid-term clinical outcomes^3^, which have recently gained importance with respect to TAA.

Despite functional benefits, results after TAA have not reached the same level as those for hip and knee arthroplasty^4^. Using meta-analysis, the risk of reoperation with the use of TAA was significantly higher than that with the use of ankle arthrodesis (risk ratio [RR] = 1.81; 95% confidence interval [CI], 1.37–2.39)^5^. According to a recently published meta-analysis, pooled proportion of conversion to arthrodesis or revision at 5 and 10 years of minimum follow-up was 0.122 (95% CI: 0.084–0.173)–0.185 (95% CI: 0.131–0.256) and 0.202 (95% CI: 0.118–0.325)–0.305 (95% CI: 0.191–0.448), respectively^2^.

Although studies have identified some risk factors including age, sex, race, type of implant, and radiologic findings associated with the failure of TAA with small cohorts, the number of studies is relatively small compared to studies on other joint replacement techniques. Previous cohort studies also have some limitations. Different databases use different methods of data collection, and the answer to a given study question may vary according to the database used as they represent different populations^6^. In addition, lack of various comorbidities as study variables would limit their usefulness. To improve the outcomes of future patients, it is necessary to carefully analyze the modifiable risk factors for failure. Furthermore, the absence of a clear definition of revision^7,8^ or failure^9^ may lead to underestimation or overestimation of failure.

Almost all people in South Korea are covered by a single national health insurance program, and we were able to collect data from the insurance claims database that involved a large cohort that underwent a TAA procedure. The aim of the present study was to calculate the early failure rate and to identify patient factors associated with the early failure of TAA.

## Materials and methods

### Dataset

The Ethics Committee of Hallym University (HALLYM 2020-02-009) approved the use of the data from the insurance claims database. Written informed consent was waived by the Institutional Review Board (The Ethics Committee of Hallym University). All methods were performed in accordance with the relevant guidelines and regulations. This is a retrospective cohort study using data from the Korean Health Insurance Review and Assessment Service (HIRA). About 97% of the entire population enrolled in the South Korean National Health Insurance (NHI) program and another 3% enrolled in the Medical Aid Program. The claims data for reimbursement of medical costs of these patients are submitted to the HIRA. Healthcare providers submit their patients’ data to the HIRA, which includes the diagnosis code according to the International Statistical Classification of Diseases and Related Health Problems, 10th revision (ICD-10), procedure codes, prescriptions, medical costs, and other demographic data, including patient age, sex, hospital admissions, insurance type, and comorbidities. All data from the HIRA are anonymous and encrypted to protect participants’ privacy.

### Study design and participants

**Figure 1:**
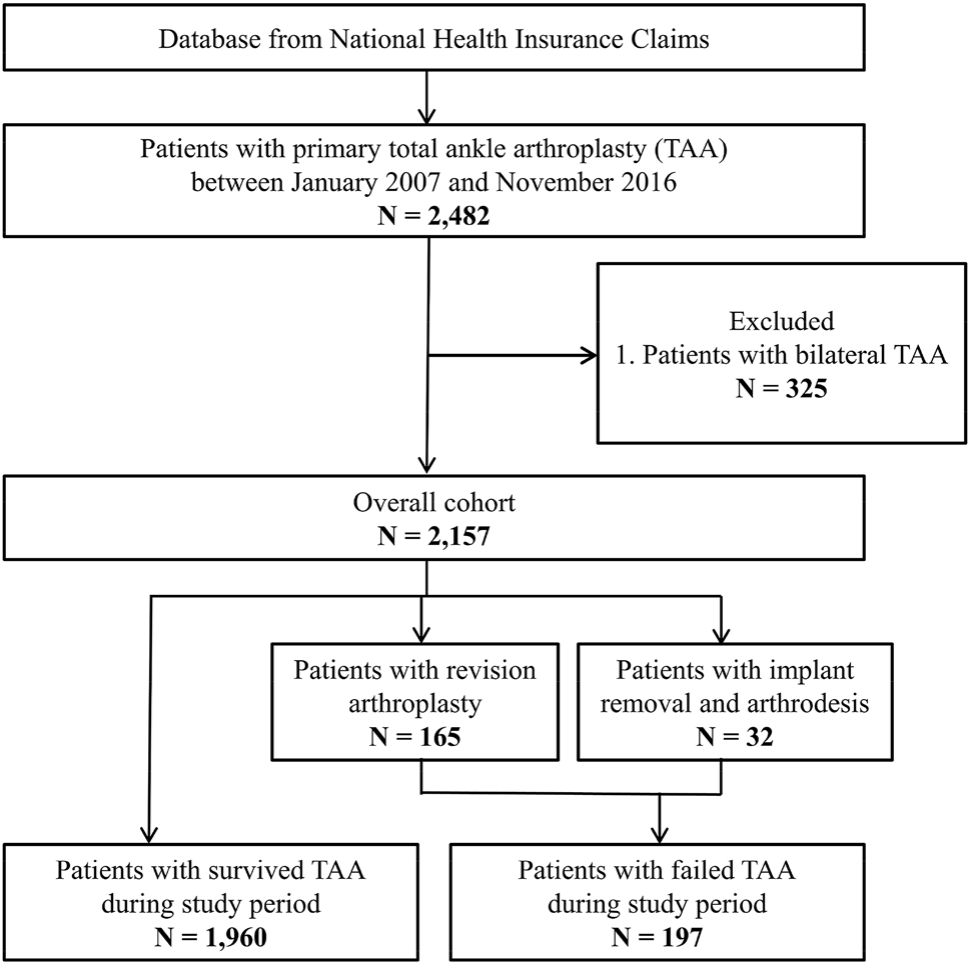
A schematic illustration of the participant selection process that was used in the present study. Out of a total of 2482 participants, 2,157 total ankle arthroplasty (TAA) participants were selected.

We included patients who have TAA procedure codes (Electronic Data Interchange [EDI]: N0279 or N0279) from 2007 to 2016. A flow diagram of the inclusion of patients is shown in Fig. 1. All included patients had a minimum of 1-year clinical follow-up, and patients who underwent a bilateral TAA procedure during the study period were also excluded. Early failure was defined as TAA requiring revision arthroplasty or implant removal and arthrodesis after primary TAA within five years. The duration of early failure was based on previous studies. The failure was reported to occur at an average of 16.4 months^7^, and the mean implant survival time to revision for any cause was reported to be between 48 and 86 months^2^. Patients who were followed up for at least five years without revision or implant removal and arthrodesis procedure after index primary TAA procedure were defined as the “No failure” group. Patients who underwent revision arthroplasty or implant removal and arthrodesis procedure within 5 years after index primary TAA procedure were defined as the “Failure” group. Patients were identified by procedure codes: TAA revision as EDI N3715 or N3719 or EDI N4715 or N4719. For ankle arthrodesis, patients who have both TAA implant removal code and ankle arthrodesis code were identified: TAA implant removal as EDI N3725 or N3729 and ankle arthrodesis as EDI N0733 or N0736. In this case, the date of ankle arthrodesis was followed by the primary TAA date.

### Covariates

The covariates included in the present study included age, sex, admission, duration, and comorbidities. Comorbidities of patients were identified based on a previously published protocol^10,11^. We included myocardial infarction (ICD-10: I21 or I22), congestive heart failure (ICD-10: I50), cerebrovascular disease (ICD-10: I60-I69), chronic pulmonary disease (ICD-10: J44), hypertension (ICD-10: I10-I15), diabetes mellitus (ICD-10: E10-E14), renal failure (ICD-10: N17–N19), rheumatoid arthritis (ICD-10: M058, M059, M068 or M069), peripheral vascular disease (ICD-10: I73), hyperlipidemia (ICD-10: E78), dementia (ICD-10: F00 through F03), obesity (ICD-10: E66), psychosis (ICD-10: F20–F29), depression (ICD-10: F33), osteoporosis (ICD-10: M80–M84) and alcohol abuse (ICD-10: F10)^10,11^. All the comorbidities were included as patient risk factors if they were diagnosed before the index primary TAA procedure.

### Statistical analysis

All continuous variables are expressed as means and standard deviation (SD). We used Pearson’s chi-squared test to compare qualitative differences. Fisher’s exact test was performed if the number of expected cases was less than five. The significance of differences in continuous variables was explored using the independent samples t-test. We employed multivariate logistic regression to identify prognostic risk factors for early failure. The selected variable for multivariate analysis included variables with a *p* value < 0.2 in univariate analysis. The odds ratios (OR), 95% confidence intervals (CI), and *p* values of various patient demographic characteristics and comorbidities were calculated. A *p* value < 0.05 indicated statistical significance. All statistical analyses were performed using SPSS for Windows software (ver. 16.0, IBM Corp., Armonk, NY, USA) and SAS Enterprise software (version 6.1: SAS Institute Inc. Cary, NC, USA).

## Results

Among 2482 patients who underwent TAA and who were invited for the current study, 2157 patients who underwent TAA were selected for the final analysis (Fig. 1). During the study period, 197 patients developed early failure, for an overall early failure rate of 9.1%. Among them, 165 patients (84%) required revision arthroplasty and 32 patients (16%) required implant removal and arthrodesis.

The results of the univariate analysis for the comparison of the two groups are listed in Table 1. History of chronic pulmonary disease (*p* < 0.001), diabetes (*p* < 0.001), peripheral vascular disease (*p* = 0.005), hyperlipidemia (*p* < 0.001), dementia (*p* < 0.001), and alcohol abuse (*p* = 0.002) showed significant differences between the two groups.

**Table 1:** Comparison between two groups.

	No failure group	Failure group	*p* value
Number of patients	1960	197	
Age (mean, standard deviation)	69.3 (9.72)	66.7 (10.25)	0.07
Sex (% of female)	959 (48.9%)	93 (47.2%)	0.65
Admission duration (day)	17.3 ± 11.27	16.4 ± 10.3	0.30
**Comorbidities**			
Myocardiac infarction	156 (8.0%)	9 (4.6%)	0.09
Congestive heart failure	144 (7.4%)	21 (10.7%)	0.09
Cerebrovascular disease	297 (15.2%)	40 (20.3%)	0.06
Chronic pulmonary disease	232 (11.8%)	45 (22.8%)	< 0.001
Hypertension	1053 (53.7%)	112 (56.9%)	0.40
Diabetes	583 (29.7%)	93 (47.2%)	< 0.001
Renal failure	37 (1.9%)	7 (3.6%)	0.11
Rheumatoid arthritis	283 (14.4%)	37 (18.8%)	0.10
Peripheral vascular disease	498 (25.4%)	68 (34.5%)	0.005
Hyperlipidemia	806 (41.1%)	112 (56.9%)	< 0.001
Dementia	27 (1.4%)	9 (4.6%)	< 0.001
Obesity	25 (1.3%)	5 (2.5%)	0.15
Psychosis	87 (4.4%)	11 (5.6%)	0.46
Depression	166 (8.5%)	20 (10.2%)	0.42
Osteoporosis	861 (43.9%)	91 (46.2%)	0.54
Alcohol abuse	151 (7.7%)	28 (14.21%)	0.002

*Chi-square test or Fisher’s exact test. Significance at *p* < 0.05.

**Figure 2:**
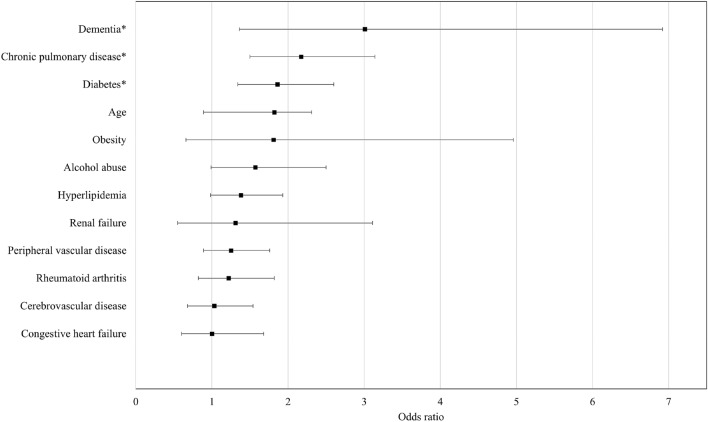
Odds ratio by multiple logistic regression analysis. The asterisk (*) indicates significant high OR.

Using multivariate logistic regression analysis, dementia showed the highest significant OR (3.01, 95% CI 1.36–2.60, *p* = 0.006) for early failure of primary TAA (Table 2). Additionally, significantly high OR were seen in patients with a history of chronic pulmonary disease (OR 2.17, 95% CI 1.50–3.14) and diabetes for early failure of primary TAA (OR 1.86, 95% CI 1.34–2.60) (Fig. 2).

**Table 2:** Results of multiple logistic regression analysis.

	Odds ratio	95% Confidence interval	*p* value
Dementia	3.01	1.36 to 6.92	**0.006**
Chronic pulmonary disease	2.17	1.50 to 3.14	** < 0.001**
Diabetes	1.86	1.34 to 2.60	** < 0.001**
Age	1.82	0.89 to 2.31	0.125
Renal failure	1.31	0.55 to 3.11	0.542
Rheumatoid arthritis	1.22	0.82 to 1.82	0.324
Peripheral vascular disease	1.25	0.89 to 1.76	0.203
Obesity	1.81	0.66 to 4.96	0.252
Hyperlipidemia	1.38	0.98 to 1.93	0.065
Cerebrovascular disease	1.03	0.68 to 1.54	0.898
Congestive heart failure	1.00	0.60 to 1.68	0.996
Alcohol abuse	1.57	0.99 to 2.50	0.054

Bold values indicate statistically significant findings.

*Chi-square test or Fisher’s exact test.

Significance at *p* < 0.05.

## Discussion

The principal finding from this nationwide cohort study with 2,157 patients who underwent TAA was that the risk of early failure seems to be higher for patients with the following comorbidities: chronic pulmonary disease (*p* < 0.001), diabetes (*p* < 0.001), peripheral vascular disease (*p* = 0.005), hyperlipidemia (*p* < 0.001), dementia (*p* < 0.001), and alcohol abuse (*p* = 0.002). Furthermore, dementia, chronic pulmonary disease, and diabetes showed increased OR of 1.86–3.01 in multivariate logistic regression analysis.

To our knowledge, this study is the largest cohort study for the evaluation of the early failure risk of primary TAA. Because it is mandatory for all Koreans to enroll in the National Health Insurance Service, this cohort data contains information on almost all Korean people who underwent a TAA procedure, and exact population statistics can be determined using this database.

In our study with 2157 patients, the early failure rate after primary TAA was 9.1%. The reported survival rate is higher than 78% of literature reviews^12^ and similar to 81–92.9% of recent studies^4,7,13,14^. The difference between the early failure rate and overall survival rate may have influenced the inconsistent results. Poorer results from earlier generations of prosthesis will have a negative effect on the overall survival and revision rates^13^. Among the registry reports, the 5-year survival rate (total cases, year of analysis) was 78% in the Swedish registry (1296 TAA, 1993–2005), 84.5–90.5% in the Australian registry (2272 TAA, 2008–2018), 90.8% in the New Zealand registry (1619 TAA, 2000–2018), and 93.14% in the National Joint registry for United Kingdom (5587 TAA, 2010–2018). The variations in the registry designs, the study populations, and the failure of accurate recording could explain the conflicting results of prior studies^13^.

Patient risk factors for early revision were already studied in total hip arthroplasty (THA) and total knee arthroplasty (TKA)^15^ and can be extrapolated. Diagnoses of diabetes, chronic pulmonary disease, dementia, Parkinson’s disease, lung circulation disorders, neurological diseases, alcohol abuse, drug abuse, depression, obesity, and fluid/electrolyte disorders significantly increased the risk for early revision of THA^16,17^. In another study, the revision rates were higher among patients with hypertension and those with paraparesis/hemiparesis for THA and among patients with metastatic disease for TKA^18^. About THA dislocation, a history of spinal fusion, Parkinson's disease, dementia, depression, and chronic lung disease were significantly related^19^.

Previous literature described that with TAA, higher comorbidity was not associated with readmission^20^, failure^14^, or revision^21^. However, the adverse impact of comorbidities on TAA complication has been previously documented by several authors^22,23^. Previous studies measured comorbidity with Deyo-Charlson index, without differentiating each comorbidity^7,14,21^. In addition, there are few large series in which all the comorbidities have been assessed concomitantly^20,24^.

Although statistically influential factors were observed, the evidence regarding dementia and chronic obstructive pulmonary disease (COPD) could not be found in literature concerning TAA; therefore, studies on TKA and THA were referred to instead. Previously, dementia was not a risk factor for periprosthetic joint infection following TKA^25^ or THA^26^. However, patients with Alzheimer’s disease and dementia were a risk factor for revision or dislocation after THA^19,27–29^. In a systemic review article, decreased cognitive function scores were associated with a functional decline after TKA^30^. Dementia is an independent risk factor for experiencing a serious injury related to a fall^31^ and a risk factor for hip fracture even at the early stage^32^. This characteristic in dementia patients may also influence the outcome of TAA.

Multiple studies have studied the impact of COPD on outcomes in lower limb arthroplasty. Patients with COPD were not at an increased risk of developing wound complications after lower limb arthroplasty^33^. In other studies, COPD was a predictor of surgical site infection after TKA^34^ and THA^35^. In addition, COPD was a risk factor for revision of TKA within 12 months^36^ and revision of TKA in 10 years^37^. Patients with COPD frequently have many comorbidities, and other comorbidities may have influenced the outcome^34,35^.

Diabetes is known to be a relative contraindication of TAA^38^. No statistical difference in secondary operations, revisions, or failure rates between the diabetes and control groups were observed in a study of TAA patients^38^. However, diabetes was a risk factor for infected TAA^39^ or failure of TAA^40^, and the chance of implant survival was higher and rate of early onset osteolysis was lower in the non-diabetic group^41^.

The effect of age on the surgical result of primary TAA has been studied with various results. There are inconsistencies among studies on the effect of age on the survival of TAA. Several studies with small sample sizes reported no significant association between age and revision^40,42^. In contrast, the survival rate of patients who underwent TAA was lower in younger patients^14,43^. This may be explained by the fact that younger patients have higher demands and activity levels than older patients^43^. Interestingly, older age groups had a higher OR of length of hospital stay but a lower OR of in-hospital infection in a cohort study on TAA^21^. As recent trends demonstrate an increase in TAA, concerns about implant survivorship persist, as long-term follow-up is needed and options for revision remain limited^44^.

Other comorbidities were not found to correlate with an increased risk of early failure of TAA in this study. Meanwhile, patients with rheumatoid arthritis showed a significantly increased risk of TAA failure^7^. The incidence of revision TAA was significantly higher in obese patients than in nonobese patients^24^. The nonobese and obese cohorts were significantly different in medical comorbidities including diabetes, obstructive sleep apnea, hyperlipidemia, hypertension, congestive heart failure, coronary artery disease, chronic kidney disease, COPD, and chronic liver disease. Increased long-term risk of TAA failure among obese patients has been reported^45^. Recently, the clinical outcomes after TAA were poorer in patients with depressive symptoms^46^. Geographic variability and heterogeneity in THE definition of comorbidities may be key factors regarding differences in reported data.

There were several strengths in the present study. This study was based on an extremely large national population, while many previous studies included only a small number of patients. Previous studies were less clear about the inclusion criteria for study patients and did not analyze potential confounders such as age, sex, and comorbidities^15^. Because the NHI data include all national citizens without exception, there were no missing participants. Furthermore, strict inclusion criteria to define failure of TAA were used to minimize variation. Lastly, registry data include a wide range of data from surgeons, and that gives the picture of the real-life situation of foot and ankle surgeons instead of specialized surgeons or units.

Our study also had some limitations. First, it includes the inherent bias of a retrospective analysis as well as inaccuracies in coding. Scant evidence is present regarding this, thus further research is needed. Second, the current database did not include the choice of implant and the inclusion of different implants. This may influence the result as reported by Hintegra, which showed lower implant failure (adjusted OR 0.31, 95% CI 0.15–0.66; *p* = 0.002) with the Mobility implant^47^. However, most of the instruments used in Korea are Hintegra or Salto, and the implant may not have had a significant effect on the outcome. Third, variables including alignment of the joint and postoperative patient-reported outcome measures could not be accounted for in a retrospective cohort study with national insurance claims data. Although these limitations are important, we believe that the large sample size of the database can provide valuable information. It is mandatory that future studies include re-analysis of TAA to evaluate long-term survival rates in patients with advanced prosthetic designs^14^. Finally, as the general code did not further specify where TAA was performed within the ankle, patients who underwent TAA on both sides were excluded from this study. In addition, there can be patients with poor outcomes who chose not to undergo revision^48^, although that number is expected to be small. Such variables for each patient were not studied and should be addressed in future studies.

## Conclusion

According to previously published research, the present study supports the importance of comorbidities in predicting early revision of TAA. In conclusion, this study demonstrated that patients with dementia, chronic pulmonary disease, and diabetes were more likely to show early failure of TAA. We believe that understanding comorbidities may provide valuable clinical insight in predicting the potential risk for TAA failure.

## Data Availability

The data of this study are available from NHIS, but restrictions apply to availability. These data were used under license for the current study only and are not publicly available.
